# Distinct patterns in study techniques and intrinsic challenges in nursing students’ self-directed learning: an exploratory, comparative qualitative study

**DOI:** 10.1186/s12909-026-09892-3

**Published:** 2026-07-09

**Authors:** Monika Ravik, Charlotte Weider, Hanne Nørbygaard Bjerva, Karin Bölenius

**Affiliations:** 1 Department of Nursing and Health Sciences, Faculty of Health and Social Sciences, University of South-Eastern Norway, Post-box 4, Borre, 3199 Norway; 2 Karin Bölenius Faculty of Medicine, Department of Nursing, Umeå University, Umeå, 901 87 Sweden

**Keywords:** Anatomy, Biochemistry, Learning, Nursing education, Nursing students, Physiology, Self-directed learning

## Abstract

**Background:**

Self-directed learning supports nursing students’ professional development by enabling them to deepen their theoretical knowledge, particularly in complex foundational subjects such as anatomy, physiology, and biochemistry. Becoming a self-directed learner is therefore crucial for academic success and continued professional growth. However, intrinsic (student-related) factors can challenge students’ efforts to engage in effective self-directed learning, especially in these demanding subjects. Despite this, little is known about which intrinsic factors first-year nursing students perceive as hindering their self-directed learning in biosciences, and whether these differ between younger and mature students. Understanding these intrinsic factors in detail is thus essential for nurse educators who support nursing students’ learning in anatomy, physiology, and biochemistry. The aim of the study was to explore and compare intrinsic factors that challenge young and mature nursing students’ engagement in self-directed learning of anatomy, physiology, and biochemistry.

**Methods:**

An exploratory qualitative design was employed. Written responses from 98 first-year nursing students were collected in November 2024. Each response was approximately 150 words in length. The students answered an open-ended question about contextual and intrinsic factors hindering self-directed learning in anatomy, physiology, and biochemistry, submitted via the university’s digital learning platform. The responses were analysed using qualitative content analysis, with a comparative component between two age-defined groups (younger and mature students).

**Results:**

Four categories of intrinsic factors that challenge first-year nursing students’ self-directed learning in anatomy, physiology, and biochemistry were identified: (1) Challenges with effective study techniques in self-directed learning, (2) Challenges with motivation in self-directed learning, (3) Health-related constraints on self-directed learning, and (4) Procrastination in self-directed learning.

**Conclusions:**

The results revealed intrinsic factors that challenged self-directed learning in anatomy, physiology, and biochemistry among nursing students. The results underscore the need for nurse educators and institutions to recognize and address the intrinsic factors when designing learning environments and support strategies, in order to better facilitate effective self-directed learning in foundational subjects in nursing.

**Supplementary Information:**

The online version contains supplementary material available at https://doi.org/10.1186/s12909-026-09892-3.

## Contribution of the paper

### What is already known

- Self-directed learning is a dynamic, reflective process where learners autonomously plan, manage, and evaluate their learning, and is a professional necessity in nursing.
- Bioscience subjects are essential but demanding, requiring extensive self-directed learning beyond scheduled teaching.
- There is limited knowledge about intrinsic factors hindering first-year nursing students’ self-directed learning in biosciences, especially across age groups.

### What this paper adds

- Nursing students experience intrinsic self-directed learning challenges in biosciences.
- Younger students are particularly affected by inadequate motivation.
- Mature students particularly struggle with inadequate self-directed learning strategies.

## Introduction

Self-directed learning (SDL) is a process in which learners take primary responsibility for planning, implementing, and evaluating their own learning [1, 2]. SDL has gained particular importance in higher education, especially in nursing and other health professions, where it underpins lifelong learning and evidence-based practice [3]. Rapid advances in biomedical knowledge and frequent updates to clinical guidelines require practitioners to continually maintain and expand their competencies. In this context, SDL is increasingly viewed as a professional necessity rather than merely a pedagogical option [4].

More specifically, SDL can be understood as a form of learner autonomy—that is, the extent to which students decide what, how, and when they learn [2]. This autonomy depends on effective self-management, including organizing time, selecting and using appropriate resources, and applying strategies that support goal attainment [1, 2]. In addition, SDL involves self-monitoring and self-evaluation, whereby learners reflect on their understanding, performance, and progress, and adjust goals, strategies, or effort as needed [2]. Together, these elements depict SDL as a dynamic, reflective approach in which learners actively steer and regulate their own learning processes.

In nursing education, students are expected to engage in SDL in, for example, foundational bioscience subjects such as anatomy, physiology, and biochemistry [5]. These subjects are often perceived as cognitively demanding and require substantial independent study outside scheduled teaching [6]. At the same time, nursing students represent a heterogeneous group with varying educational backgrounds, life experiences, and life circumstances—factors that may influence how they engage with SDL [7].

SDL is influenced by a range of factors that can be broadly divided into intrinsic (student-related) and contextual aspects [8]. In this manuscript, we use the term intrinsic factors to refer to characteristics and processes within the student, encompassing their personal dispositions and internal learning resources. By contrast, contextual factors refer to contextual demands. The present study focuses on intrinsic challenges with SDL, while contextual demands have been explored in a separate companion study [9]. Despite the central role of SDL in nursing education, it remains comparatively underexplored [10]. In particular, the intrinsic challenges associated with SDL in biosciences, and how such challenges are experienced by first-year nursing students of different age groups, are insufficient explored [11] .

In light of this, the present study focuses on young and mature nursing students’ experiences of intrinsic factors that challenge their engagement in self-directed learning for subjects such as anatomy, physiology, and biochemistry.

## Background

As they transition to university, first-year nursing students often demonstrate high intrinsic motivation [12, 13], driven by the novelty of the learning environment, new academic demands, and a strong interest in their chosen profession [14]. This initial motivation is particularly important as students commence foundational bioscience courses, such as anatomy, physiology, and biochemistry [15]. These first-year subjects provide essential scientific foundations for nursing education, underpinning later clinical learning and safe evidence-based patient care [16–18]. Nevertheless, many nursing students experience these subjects as challenging, and failure rates in related examinations remain high [6, 19, 20].

The bioscience curriculum is extensive and conceptually demanding, requiring sustained, in-depth study [21]. Although lectures, seminars, and practical sessions provide essential frameworks, they rarely cover the content in sufficient breadth and depth [1, 20]. Consequently, nursing students must engage with the material beyond scheduled teaching and take initiative in their own learning [1, 22, 23]. In this context, SDL becomes particularly important, as it enables nursing students to take control of their learning pathways in these complex subjects [1, 2, 22].

For nursing students, SDL can deepen understanding of complex bioscientific content and support the development of effective and competent nursing practice [2, 12, 22]. Existing research indicates that nursing students are capable of learning autonomously [11–13, 24]; however, most studies have focused on students in later stages of their bachelor programme [11, 13, 24]. Ravik et al. [9] included initial nursing students, but concentrated on contextual demands rather than intrinsic challenges in the learning of anatomy, physiology, and biochemistry. Hence, there is still limited knowledge about intrinsic factors that hinder first-year nursing students’ SDL of biosciences [1, 9].

Younger nursing students may find SDL particularly challenging, often due to limited experience in planning, managing, and evaluating their own learning [1, 7, 25]. Many enter nursing programmes directly from highly structured, teacher-led, and closely supervised school environments [26]. The transition to higher education—requiring greater autonomy, self-regulation, and responsibility— can therefore feel abrupt and overwhelming [25, 26]. In contrast, little is known about how mature nursing students engage in SDL, and their experiences remain underrepresented in the literature [27, 28]. The term “mature student” generally denotes an individual who returns to education later in life, typically following a period away from formal study [29]. Future research should examine SDL in nursing education with explicit attention to age-related differences [13, 15, 19, 27, 29, 30]. This knowledge can inform tailored pedagogical and support interventions to strengthen SDL competence [11, 25, 31]. For younger students, this may include structured guidance in goal-setting, time management, and reflection; for mature students, support might focus on integrating SDL that recognizes prior learning, and using life experience as a resource. By aligning teaching strategies with the distinct needs of younger and mature students, educators can more effectively foster SDL competencies, enhance academic performance, promote lifelong learning, and better prepare students for the complex demands of professional nursing practice [3, 32].

This study explores and compares intrinsic factors that challenge young and mature nursing students’ engagement in SDL of anatomy, physiology, and biochemistry.

The following research question guided the study:

- Which intrinsic factors challenge nursing students’ engagement in SDL in anatomy, physiology, and biochemistry across different age groups?

## Methods

### Study design

An exploratory qualitative study using qualitative content analysis was conducted to explore intrinsic factors that challenge first-year nursing students’ SDL in the biosciences. An exploratory design is suitable to enabling in-depth exploration and when knowledge is limited [33]. Qualitative content analysis is a systematic research approach that is used to interpret and organize qualitative data by identifying, categorizing, counting, and examining patterns in words, themes, and concepts, as well as the relationships between them [33, 34].

This study is part of a larger research project on learning processes in anatomy, physiology, and biochemistry in nursing education (Project number 815027). The dataset comprises 98 written responses and has also been used in a companion study [9] that focused on contextual demands challenging nursing students’ SDL. In the present study, the focus was on intrinsic, student-related factors, providing a complementary perspective on the shared dataset. Although the data are shared, different meaning units were selected and analysed in the two studies to address these distinct aspects of SDL. We recognise that the analytic distinction between “intrinsic” and “contextual” is heuristic, and that these domains are closely intertwined. Our focus on intrinsic factors should therefore be understood as a deliberate, but partial, lens on the broader set of influences on students’ SDL [9]. No identical quotations or categories are reported in both papers. Each manuscript has its own analytic focus and set of categories, ensuring that the two studies provide complementary, rather than overlapping, analyses of the shared dataset.

### Study sample and recruitment

The study was conducted at a university in the southern part of Norway offering a three-year bachelor programme in nursing (European Credit Transfer and Accumulation System). A convenience sampling strategy with invited total cohort participation was used [35]. All first-year nursing students who were enrolled in the bachelor programme in nursing at the study site, were in their first semester and were preparing for their first attempt at the examination in anatomy, physiology, and biochemistry were invited to participate.

After receiving approval from the Vice-Dean of the Faculty of Health Sciences, all eligible first-semester nursing students (*N* = 147) at the study site were invited to participate via the university’s digital learning platform. To be included, students had to be enrolled in the first semester of the nursing programme and be preparing for their first attempt at the bioscience examination. Students who were retaking the exam because of earlier failures were excluded. Of the 147 invited students, 98 consented to participate and submitted one written response each. Given the focused aim, the relatively homogeneous educational setting, and the generally rich content of the responses, we judged this sample to provide sufficient information to address the research question [36].

Age data were collected to enable stratification of participants into two age groups for analytical comparison. In this study, we defined a “mature student” as a student aged 26 years or older. There is no universally agreed definition of a mature student, but previous research often describes mature students as those who enter or return to higher education later than the typical school-leaver, frequently after a period in work or with family responsibilities [29]. In our context, students aged 26 and above were more likely to have had a break from formal education and to have accumulated such experience. We acknowledge that this cut-off is partly pragmatic and that other thresholds could have been chosen; our age-based comparisons should therefore be interpreted as patterns within this sample rather than definitive age-based differences. On this basis, the younger student group (Group A) comprised 69 students aged 19–25 years (mean age 20.7 years), and the mature student group (Group B) comprised 29 students aged 26–47 years (mean age 34.3 years) (Table 1). These background differences are likely to shape how students experience and manage intrinsic challenges in SDL and therefore informed our comparative analysis [37, 38].

**Table 1:** Overview of the number of written responses containing categories reflecting intrinsic challenges in SDL. Characteristics of the two student groups by numbers of participants and age

Student characteristics	Student group A	Student group B
The number of students	69 students	29 students
Age	19–25 years	26–47 years
Mean age	20.7 years	34.3 years

Age data were collected to explore potential age-related patterns regarding similarities and differences. Comparing these groups supports a more nuanced understanding of each group’s specific needs, challenges, and strengths in relation to intrinsic challenges [30, 35].

### Data collection

Data were collected in November 2024 using a single, researcher-generated open-ended question administered via the university’s digital learning platform. All eligible students accessed the question through the platform and were asked to submit an approximately 150-word written narrative response.

The prompt was: “What factors prevent you from dedicating sufficient time to self-directed learning in anatomy, physiology, and biochemistry?”

This open-ended prompt was a study-specific item developed for the broader research project on learning processes in biosciences. The question was informed by the literature on SDL in nursing education and the aim to identify perceived barriers to SDL. The wording was discussed within the research team to ensure clarity and relevance.

Students submitted their written response electronically via the learning platform within a specific time frame. Each response was treated as an individual narrative describing the students’ perceived intrinsic challenges related to SDL in biosciences.

### Data analysis

Qualitative content analysis as described by Graneheim and Lundman [39] was used to systematically categorize and interpret the textual data, thereby enabling the identification of patterns and categories relevant to the research question [39]. A complementary numerical component was included to count elements such as category distribution, thereby broadening and reinforcing the qualitative results [34].

Each student’s written response was read several times by three authors (MR, HNB, CW) to gain an overall sense of the material and to establish a shared understanding of the data. The first author (MR) then conducted the initial analysis by coding the data inductively. Each written response was treated as a unit of analysis but often contained several distinct meaning units, and thereby coded aligned with the study aim.

A coding scheme (Supplementary File 1) was developed on this basis. All coding was performed manually. Inductive coding, in which codes emerge directly from the data rather than from a predefined framework, allowed new insights and patterns to be identified [40]. The second (CW) and third (HNB) authors then reviewed and validated the initial coding framework and carefully examined the material for additional codes. No new codes were identified, which supports the comprehensiveness of the initial coding. This collaborative process enhanced the trustworthiness of the coding. Irrelevant text that did not address the study aim was excluded to maintain focus on intrinsic challenges in SDL.

Related codes were then organized into higher-order categories. During this stage, similar codes were merged and redundant codes were removed. Identifying relationships among codes facilitated the organization of the data into broader, analytically meaningful categories [35]. Consequently, a single response could be represented in more than one category if the student described different types of intrinsic challenges (for example, both motivation and study techniques) within the same text.

After the categories had been established, we conducted a comparative analysis of their distribution across the two age-defined groups. We counted how many written responses in each group contained each category and calculated simple percentages. This provided an overview of how each category was represented in each student group and supported the identification of patterns [34]. In line with Sandelowski’s [34] argument on the cautious use of numbers in qualitative research, these numerical summaries were used solely to illustrate the relative distribution of categories within the sample and to enhance transparency about how often particular patterns appeared in the data. The core of the analysis and interpretation remained qualitative and focused on the content and meaning of participants’ accounts, with the numerical counts serving as a complementary aid rather than as a basis for statistical comparison or claims of importance. This comparative analysis highlighted similarities and differences in intrinsic challenges in SDL across the two student groups. Appropriate quotations were identified.

In this study, attention-deficit/hyperactivity disorder (ADHD) and dyslexia are reported only when participants themselves explicitly referred to having a formal diagnosis in their written responses (e.g., “I have been diagnosed with ADHD…”). We did not diagnose ADHD or dyslexia, nor did we infer ADHD or dyslexia from general concentration difficulties, fatigue, or short reading span. Such difficulties are described in more neutral terms (e.g., “concentration difficulties”) unless participants explicitly linked them to ADHD or dyslexia. Thus, ADHD and dyslexia are treated as a self-reported diagnosis, as understood and described by the participants themselves.

### Trustworthiness

We applied Guba and Lincoln’s [41] trustworthiness criteria to ensure that the research was conducted rigorously and transparently. By addressing credibility, dependability, transferability, and confirmability, the study’s trustworthiness was strengthened.

Credibility was supported by a transparent description of the data collection tool and process, and by a systematic analytic process. The open-ended prompt was designed and discussed within the research team to elicit rich, narrative accounts directly relevant to the research question. All 98 responses were read repeatedly by three authors to achieve immersion, and the subsequent qualitative content analysis followed the stepwise phases, including collaborative coding and refinement of categories.

Dependability was enhanced by providing detailed descriptions of the study context, sampling and recruitment strategies, the data collection tool, and each step of the analysis. This allows readers to follow how data were generated and transformed into categories and comparative patterns.

Transferability was addressed by offering a thick description of the educational context, the study site, participant age characteristics, and the nature of the data (brief written responses to a single open-ended question). We also acknowledge, in the Limitations section, factors that constrain transferability, such as the single-site Norwegian context and the use of limited demographic information.

Confirmability was supported through reflexive and collaborative analysis. The first author conducted the initial coding, while the second and third authors reviewed and discussed the coding scheme, category boundaries, and comparative interpretations. This team-based approach, together with the clear documentation of the data collection tool and analytic decisions, helped to minimise individual bias and strengthen the transparency of the results.

### Ethical considerations

The study was conducted in accordance with national and institutional ethical guidelines [42]. Approval was obtained from the Norwegian Agency for Shared Services in Education and Research (project No. 815027) and from the participating university (the Head of the Faculty of Health and Social Sciences) prior to data collection. In line with national regulations, separate ethics committee approval was not required.

Participation was voluntary and clearly separated from course requirements and assessment. Students received written and oral information about the study’s purpose, the use of their written responses, and their right to withdraw at any time without consequences. Those who agreed to take part provided written informed consent [42].

To safeguard confidentiality, all identifying information was removed from the written responses, and each response was assigned a unique code. Age was retained only at group level to enable the comparative analysis. Data were stored securely and were accessible only to the research team. The written responses were used solely for research purposes.

In reporting health-related information, we took particular care to avoid overinterpretation. ADHD and dyslexia were mentioned only when students themselves explicitly referred to having a formal diagnosis in their written responses (e.g., “I have been diagnosed with ADHD…”). We did not infer ADHD from general concentration or reading difficulties. Such information is reported only at group level and in a way that prevents identification of individual participants.

## Results

Distinct patterns in intrinsic factors influencing SDL were observed between groups. *Challenges with effective study techniques* were more frequently reported among mature students, whereas c*hallenges with motivation in SDL* were more prominent among younger students. *Health-related constraints on SDL* and *procrastination in SDL* were less commonly reported across both groups (Table 2).

**Table 2:** Overview of the number of written responses containing categories reflecting intrinsic challenges in SDL

Category	Group A: Number of written responses	Group B: Number of written responses
Challenges with effective study techniques in self-directed learning	11 of 69 (16%)	14 of 29 (48%)
Challenges with motivation in self-directed learning	22 of 69 (32%)	4 of 29 (14%)
Health-related constraints on self-directed learning	11 of 69 (16%)	4 of 29 (14%)
Procrastination in self-directed learning	4 of 69 (6%)	3 of 29 (10%)

Note: We remind readers that Group A (younger students) comprised 69 participants, whereas Group B (mature students) comprised 29 participants, and we emphasise that percentages and patterns must be interpreted in light of this imbalance

### Challenges with effective study techniques in self-directed learning

Both student groups reported challenges with effective study techniques in SDL (Table 2), but these challenges were more frequently described by mature participants (Group B) than younger participants (Group A). In the younger student group, intrinsic challenges with SDL were reported as being related to the time-consuming nature of studying anatomy, physiology, and biochemistry due to their complexity, compounded by the need to simultaneously acquire knowledge in other nursing subjects.

It is at times challenging to find the right study technique, and there are many new systems to familiarize oneself with. (Participant 1, Group A)

I am someone who takes longer to learn, which often acts as a stress factor. I can get stressed by not finishing on time and end up rushing through the topics. This can shift my focus from learning to just finishing quickly. I feel that I often need to put in double the effort to learn the same as my fellow students because I find the acquisition and processing of information challenging and, not least, time-consuming. (Participant 26, Group A)

Dyslexia was described by several of the younger participants (Group A) as a fundamental intrinsic challenge that influenced their ability to concentrate, process information, and engage with effective study techniques during SDL.

As I have dyslexia, I can take much longer to study than a ‘normal’ person would, as I struggle quite a bit with concentration and absorbing the material. (Participant 33, Group A)

I have dyslexia, which troubles me greatly and prevents me from working for as long as I would like. I get tired quickly. (Participant 39, Group A)

In the mature student group (Group B), some participants described it as challenging to engage and learn using appropriate study techniques in SDL after many years away from formal education. The fear of falling behind academically was described as being constant, as mature students described that they needed more time for SDL than their younger counterparts. They described that using inappropriate study techniques in SDL made it even more challenging to handle the large amount of material that needed to be learned simultaneously.

It takes a very long time to understand things, and I need to read and review the material many times before it sticks in my mind. Spending a lot of time on one topic, repeating it several times, means that other topics must be studied simultaneously. (Participant 87, Group B)

I no longer feel that I get anything ‘for free’. Now that I am an adult, there is not so much that comes naturally, and I notice that I have to work harder for things. (Participant 94, Group B)

### Challenges with motivation in self-directed learning

In both student groups, motivational challenges were an issue related to intrinsic SDL Motivational challenges were more frequently described by the younger student group (Group A) than in the mature student group (Group B) (Table 2). Participants in the younger student group reported that they could become unfocused, distracted, and unmotivated when faced with new and complex subject matter. They found it challenging and demotivating to work independently with complex material.

Having a lack of motivation and sufficient focus to sit with schoolwork can also be a challenge some days. There are days when I feel overwhelmed or just don’t have enough motivation to study, and it can be difficult to get through assignments when interest is low. (Participant 37, Group A)

I feel that there is a lot of curriculum, and it can be a bit overwhelming. This makes it a bit harder to get through everything and use my motivation. The subjects are very interesting, but my motivation is probably the weakest link, which makes it a bit harder to dedicate enough time to my studies. (Participant 43, Group A)

When it comes to factors that prevent me from spending enough time on my studies, the biggest obstacle is often myself. I can experience a lack of motivation and energy when I look at the tasks ahead of me. It can feel overwhelming when I know how much I have to do, and this in turn leads to feelings of exhaustion and stress. (Participant 48, Group A)

In the mature student group (Group B), some participants described a loss of motivation for SDL when they experienced doubts about their understanding and mastery of the subject matter. Despite varying levels of motivation for SDL, they mentioned that efforts were made to allocate time for it.

In addition, I often doubt my own abilities and can be unsure about whether I’ve truly understood the material. This doubt sometimes causes me to lose motivation to study as thoroughly as I should. (Participant 78, Group B)

### Health-related constraints on self-directed learning

In both student groups (Table 2), participants described health-related constraints, both somatic and mental, that negatively influenced their capacity for SDL. ADHD was identified in both groups as an important intrinsic factor that limited effective time use. Despite allocating time for SDL, participants described that they often perceived that much of the time was unproductive, as difficulties with concentration led to engagement in activities other than studying. Moreover, ADHD was reported as being associated with fatigue and exhaustion, necessitating frequent breaks and further constraining sustained engagement in academic tasks.

The real worst time thief is ADHD, so despite setting aside a lot of time for studying, only a small portion [of time] is used effectively. (Participant 19, Group A)

I have been diagnosed with ADHD as a main factor. It’s easy for others to say, ‘Just sit down and do it’. But from ADHD as a main factor, many other factors stem from it. As the day goes on, I realize that some things take longer, or I start to re-prioritize with thoughts like, ‘I know I have to do this, but I just need to…’ And ‘just need to’ is always an unconscious use of time. Another factor when I’m studying can be distractions around me. I need to have what I’m concentrating on right in front of me. For example, I can’t sit next to a window or around many other people. If a bird flies past the window, most of my concentration goes to that bird. (Participant 23, Group A)

I find it difficult to read the book because I have ADHD and have been struggling with medication and vision problems lately. I have also sometimes been so stressed over time that I have struggled with a high resting heart rate and palpitations. (Participant 83, Group B)

### Procrastination in self-directed learning

Procrastination was not a pervasive pattern for SDL in the data (Table 2), but it was more commonly perceived in the group of mature participants (Group B) than in the younger student group (Group A). In the younger student group, participants described that procrastination was a familiar intrinsic challenge related to SDL from the upper secondary school and resulted in insufficient time being spent on SDL.

It is difficult to break bad habits from high school, like procrastination. I have become better at it over time, but I feel like it still lingers, and it’s something I really need to work on. (Participant 3, Group A)

I have always been good at procrastinating. I usually put things off until the last minute, which leads to stress and often makes me delay it even more. (Participant 53, Group A)

In the mature student group (Group B), procrastination manifested somewhat differently than in the younger student group (Group A), described as a result of finding the material overwhelming. Without a concrete schedule for SDL, it was easy to prioritize other activities over studying.

I tend to struggle with procrastination. I often postpone tasks because they feel overwhelming, or I simply don’t know where to start. This leads to working under time pressure, which causes me unnecessary stress. (Participant 78, Group B)

I procrastinate so that I put off studying until it becomes too much to handle in a short time. (Participant 93, Group B)

## Discussion

This study explored and compared intrinsic factors that challenge young and mature nursing students’ engagement in SDL of anatomy, physiology, and biochemistry. The results provide insights into how, in this sample, students’ learning engagement was shaped by a range of interrelated intrinsic factors, summarized in four categories: Challenges with effective study techniques in SDL, Challenges with motivation in SDL, Health-related constraints on SDL, and Procrastination in SDL. These categories together highlight that students’ engagement in SDL is influenced by processes that extend beyond individual effort or willingness to learn. Rather, the results suggest that intrinsic challenges to SDL arise from the interaction between students’ capacities, prior learning experiences, and ability to regulate learning over time.

The present study showed that both younger and mature nursing students reported intrinsic challenges with SDL. Although some of these challenges were shared across the groups in this sample, mature students more often highlighted inadequate or "rusty" study techniques as a major challenge, whereas younger students more frequently reported low motivation. These interpretations indicate that intrinsic challenges related to SDL may be experienced somewhat differently by younger and mature students in this particular setting. However, given the exploratory design, unequal group sizes and single-site context, these patterns should be interpreted cautiously as sample-specific rather than definitive age-related differences, although previous research [1] shows that students often struggle to develop, implement, and sustain effective SDL strategies in demanding content areas such as anatomy, physiology, and biochemistry. Although SDL is promoted as a key approach for fostering autonomy, critical thinking, and lifelong learning, it presupposes intrinsic competencies such as goal-setting, planning, monitoring, and evaluating one’s own learning—competencies that cannot be taken for granted [3]. For some mature students in our study, these intrinsic demands appeared to be exacerbated by a prolonged absence from formal education. This aligns with previous research that reported that mature nursing students often perceive their study skills as “out of date”, and that re-entry into academic culture can be both cognitively and emotionally demanding [43]. Nielsen et al. [32] found that nursing students described feeling unfamiliar with contemporary academic expectations and uncertain about how to organize and regulate their learning in an SDL-oriented programme. Our results are consistent with these observations, but, given our design, we cannot determine the extent to which time since previous education is a decisive factor for all mature students. Instead, we interpret it as one possible contributor to the challenges described by some participants in this group.

Moreover, some mature students in our study appeared to face a “double burden”, which they described as a need to (re)develop SDL strategies while simultaneously engaging with a demanding bioscience curriculum. This interpretation remains cautious and is based on their self-reported experiences in this specific setting. However, bioscience is characterized by extensive terminology, abstract theoretical constructs, and the need to integrate knowledge across molecular, cellular, organ, and system levels [44], which many nursing students find overwhelming [27, 45]. In combination with underdeveloped or outdated study strategies, this perceived overload may reduce engagement and persistence in SDL, echoing earlier work that showed that demanding curricula can amplify the impact of weak study skills [9, 12].

In contrast, the younger participants in our study more often described their intrinsic SDL challenges to the volume and complexity of the curriculum rather than to a break from education. Having recently completed high school, they may have been more accustomed to being in a learning environment, although the study techniques that had been sufficient in high school were no longer adequate in higher education [46, 47]. This points to a mismatch between the learning strategies that are rewarded in high school and those that are required for successful SDL in a content-dense university programme.

An additional layer of complexity emerged for a small number of younger students who self-reported having dyslexia. Bioscience study typically demands extensive reading, note-taking, and written reflection [15]. These students described needing substantially more time to read, becoming fatigued, and finding it challenging to absorb written material during SDL. While this is consistent with descriptions of dyslexia in the literature, our study relied solely on self-reporting and did not verify diagnoses. The result should therefore not be transferred to all students with dyslexia, but should rather illustrate how some students who identify as having dyslexia experience SDL in this context. However, within an SDL framework, where students must independently plan, implement, and evaluate their learning activities [2], such difficulties can substantially undermine their capacity to assume responsibility for learning. The combination of heavy reading demands and limited institutional scaffolding of SDL processes may therefore be especially disadvantageous for this student group.

The results of this study suggest that there were age-related differences in how students talked about motivation for bioscience learning. Several of the younger students reported reduced motivation, which can be understood in light of the substantial transition from high school to higher education [47]. This transition typically involves increased academic demands, greater autonomy, and less external structure [48]. When the curriculum is experienced as overwhelming, students’ perceived competence may be threatened. From the perspective of self-determination theory [49], such experiences may undermine intrinsic motivation; however, we did not measure self-determination constructs directly, and this theoretical framing should therefore be viewed as an interpretive lens rather than as a direct empirical result. For younger students who have not yet developed robust study strategies or a strong academic self-concept, the feeling of being overwhelmed by biosciences may therefore directly undermine their motivation to engage in SDL [32].

In contrast, in this sample, fewer mature students explicitly reported low motivation. Several students described entering higher education following deliberate, reflective decisions linked to specific professional or personal goals [24]. This intentionality may provide a more stable motivational foundation, even when the programme is experienced as demanding. Previous research indicates that mature students often show higher levels of identified or intrinsic motivation, as they connect their studies to broader life projects such as career change or long-held aspirations [50]. In our data, this stronger goal orientation may help to explain why mature students, despite substantial cognitive challenges, did not report loss of motivation to the same extent as their younger peers.

Students across both age groups self-reported having an ADHD diagnosis and described this as a barrier to SDL, underscoring the role of neurodevelopmental conditions in shaping students’ capacity for autonomous learning [51]. Students reported difficulties in sustaining attention during lectures, reading, and being easily distracted, which they felt reduced the amount of time that could be used effectively for SDL. ADHD is associated with executive function difficulties in planning, time management, prioritization, and self-monitoring, all of which are central to effective SDL [52]. Our data are limited to brief, self-reported accounts and do not allow us to explore these mechanisms in depth, or to draw conclusions about ADHD more broadly or across the life course. We therefore interpret these results as illustrative of how a small number of students who identify as having ADHD experience SDL in this specific setting.

Procrastination was also identified as an intrinsic barrier to SDL by a small number of students in both age groups, and the way they described it appeared to differ somewhat. Among younger students, procrastination was often described as a continuation of habits from high school, which became more problematic in the less structured university context. This was likely a reflection of underdeveloped SDL skills that become more challenging when removed from the comparative structure of the high school context. Among mature students, procrastination was sometimes linked to feeling overwhelmed by the curriculum or unsure where to start, and to competing responsibilities outside study. These patterns are consistent with previous research that link procrastination to underdeveloped SDL skills and perceptions of overload [46]. David et al. [46] noted that students typically followed a structured routine in high school, but at university had to adjust to reduced external control, greater flexibility, and increased responsibility for SDL. However, given the limited number of accounts and the absence of in-depth interviews, we cannot determine underlying psychological mechanisms (e.g., avoidant coping) with any certainty, and such explanations should therefore be regarded as tentative. However, nursing students who received time management training grounded in SDL principles appeared better to manage their workload [53], suggesting that targeted support in planning SDL may mitigate procrastination.

### Limitations and future research directions

The use of written responses from 98 participants provided breadth and was appropriate for an exploratory qualitative study. However, this format did not allow for clarification or follow-up questions, which may have restricted our ability to explore participant’ experiences and meanings in greater detail. Interviews could have allowed more in-depth exploration of participants’ perspectives and provided opportunities to clarify and expand on their written answers. The single open-ended written prompt did not allow us to probe participants’ descriptions or understanding of ADHD and dyslexia, and future research using qualitative interviews would be better suited to explore how students with these self-reported conditions experience self-directed learning. Nevertheless, the large number of participating students and the generally rich content of their written responses [36], despite the restricted word limit, provided a robust empirical basis for the analysis and the conclusions drawn.

The use of a single open-ended written prompt may have implications for how participants framed their responses. The prompt invited participants to describe “factors” that prevented them from dedicating sufficient time to SDL, without distinguishing between contextual and intrinsic factors. This lack of nuance provided the participants with the freedom to respond in their own terms, but may also have led some of them to focus more on contextual aspects (e.g., timetable, workload, family responsibilities) and others more on intrinsic challenges (e.g., motivation, study strategies, health-related constraints). As a result, the balance between contextual and intrinsic factors in the written responses may partly reflect how the participants interpreted the question, rather than a comprehensive mapping of all intrinsic challenges. It is also possible that not all participants experienced intrinsic challenges related to SDL.

The comparative aspect of the study is constrained by the unequal sizes of the two age-defined groups (69 younger vs. 29 mature students). This imbalance limits the robustness of the group comparisons and means that the observed age-related patterns should be interpreted cautiously as indicative trends within this sample, rather than as strong differences between younger and mature students [35]. Moreover, we collected limited demographic information beyond age, which reduced our ability to explore how other factors (e.g., prior education and employment) may relate to SDL challenges. Further, participation was voluntary, introducing a potential self-selection bias, as participants who chose to respond may systematically differ from non-participants in terms of motivation, available time or experiences of difficulties. A further limitation is that the study did not explore the specific learning approaches that participants employed when engaging in SDL. Consequently, the results do not capture how participants concretely organized, processed, and applied bioscience content in self-directed contexts. Finally, the single-site design and the specific Norwegian nursing education context may limit the transferability of the results to other institutions and educational systems.

Future research should investigate specific SDL approaches in more detail to deepen the understanding of how nursing students learn biosciences and how they can be better supported in developing effective SDL strategies. In particular, in-depth qualitative interviews and longitudinal designs would allow these issues to be explored in greater depth. Such studies could provide a more detailed understanding of how intrinsic challenges related to SDL develop and change over time, how students interpret and manage self-reported conditions such as dyslexia and ADHD in their learning, and how support strategies can be tailored and adjusted as students’ progress through their nursing education.

## Conclusions

The present study shows that nursing students in both age groups experienced intrinsic challenges in engaging in SDL in biosciences, although the nature of these challenges differed. In this sample, younger students more often described motivational challenges and feelings of being overwhelmed by the volume and complexity of the bioscience curriculum, whereas mature students more frequently described challenges related to study technique and the time needed to learn after a break from formal education. A small number of students in both age groups also referred to health-related conditions, including self-reported ADHD and dyslexia, and procrastination as constraining their SDL. These age-related patterns should, however, be interpreted cautiously. The groups were unequal in size, the comparisons were based on descriptive counts rather than statistical tests, and the study was conducted at a single Norwegian university using brief written responses to a single open-ended prompt. The observed differences are therefore best understood as tentative, sample-specific patterns rather than definitive age-based distinctions. Despite these limitations, the results suggest that support for SDL in bioscience courses may need to take account of diverse student backgrounds and challenges. For younger students, this may involve targeted support for motivation and the transition to more autonomous learning; for mature students, it may include explicit guidance in (re)developing effective study techniques for a demanding curriculum. Across age groups, there appears to be a need to recognise and accommodate health-related and learning-related constraints that can affect students’ capacity for SDL.

### Implications for nursing education: towards inclusive and scaffolded SDL

Based on our results, the study has several implications for how nursing education, and bioscience teaching and learning in particular, might better support SDL. First, the results suggest that SDL can usefully be treated as a set of learnable competencies rather than an innate trait. Given that students in this sample described challenges with study technique, motivation and self-regulation, educational institutions could consider explicitly teaching SDL skills, especially in the initial phase of the nursing bachelor programme. Evidence-based strategies such as spaced repetition, retrieval practice, and concept mapping, as well as basic time management and goal-setting, may be particular helpful when they are contextualized to bioscience learning rather than presented in a generic way.

Second, our results indicate that differentiated support may be warranted. In this sample, mature students more often highlighted challenges related to study techniques and the time needed to learn, whereas younger students more frequently described motivational challenges and feelings of being overwhelmed. On this basis, mature students might benefit from orientation programmes that focus on returning to study, workshops on academic literacies, and supervision on integrating study with work and family life. For younger students, structured support for the transition from high school to university, clear articulation of expectations, scaffolded early assignments, and achievable short-term goals may help to reduce early experiences of failure and loss of motivation.

Third, the accounts of self-reported dyslexia and ADHD highlight the need for proactive and systematic support for students with learning difficulties and neurodevelopmental conditions. This may include accessible learning materials, assistive technologies, and tailored supervision in SDL strategies that compensate for specific challenges (e.g., use of planners, chunking of tasks, and shorter focused study intervals). In addition, bioscience courses that rely heavily on SDL should incorporate more structure through detailed study plans, intermediate milestones, regular formative feedback, and integrated opportunities for reflection on learning processes.

Finally, the perceived overload and time pressure in our results suggest that curriculum design itself may help ease SDL demands. Making core concepts and learning outcomes explicit, sequencing content transparently, and aligning assessments with incremental, cumulative learning could reduce the sense of overload. Such structural measures may help both younger and mature students, but are likely to be particularly important for those who enter higher education with limited or outdated study strategies, additional responsibilities, or specific learning needs. Early identification and support for students experiencing difficulties in SDL may be beneficial, both from an educational and resource perspective. Supporting students in developing more effective strategies, or in some cases, in making informed decisions about how to cope with programme demands, may contribute to more sustainable educational pathways.

Overall, the results of the present study underscore that effective SDL in biosciences cannot be achieved solely by exhorting students to “take responsibility” for their learning. Instead, they point to a need for intentional pedagogical and institutional strategies that recognize diverse student backgrounds and constraints, and that systematically scaffold the development of SDL skills across the programme.

## Supplementary Information

Supplementary Material 1.

## Data Availability

To access the data in this study, please contact the corresponding author.

## Abbreviations

SDL: Self-directed learning
