# Supplementary material for: Distinct patterns in study techniques and intrinsic challenges in nursing students’ self-directed learning: an exploratory, comparative qualitative study
Source: BMC Med Educ. 2026 Jul 9;26:1579. doi: 10.1186/s12909-026-09892-3 (PMC13637188; doi:10.1186/s12909-026-09892-3)
Supplement: Supplementary file 1 [file 12909_2026_9892_MOESM1_ESM.docx]

Supplementary File 1. Code book

Code book with overarching category, codes and explanations

| Overarching category | Codes | Explanations |
| --- | --- | --- |
| **(1) Challenges in engaging with effective study techniques** | Difficulty finding suitable methods | Struggles to identify appropriate study methods. |
|  | Limited knowledge of study strategies | Unsure which strategies are effective; time lost to trial‑and‑error. |
|  | Over‑reliance on passive reading | Mainly uses passive reading, with low perceived learning gain. |
|  | Problems with planning and time use | Limited structure and planning undermine effective study. |
|  | Dyslexia and reading/writing difficulties | Dyslexia slows reading and writing, making self‑directed work more effortful. |
|  |  |  |
|  |  |  |
| **(2) Challenges in maintaining motivation in self‑directed learning** | General lack of motivation | Interest in the subject is not enough to sustain motivation over time. |
|  | Motivation affected by pressure | Pressure reduces motivation and focus. |
|  | Fluctuating motivation over time | Motivation varies, leading to inconsistent study effort. |
|  | Motivation undermined by study difficulties | Experiencing low mastery feeds into reduced motivation. |
|  |  |  |
|  |  |  |
| **(3) Health‑related constraints influencing self‑directed learning** | Physical health problems | Somatic health issues limit capacity for sustained study. |
|  | Mental health challenges | Mental health interferes with concentration and stamina. |
|  | Fatigue / low energy | Persistent tiredness makes self‑directed learning difficult. |
|  | Neurodiversity (ADHD) | ADHD‑related challenges disrupt reading, focus, and regulation. |
|  |  |  |
|  |  |  |
| **(4) Procrastination patterns undermining sustained self‑directed learning** | Postponing start of study sessions | Persistent habit of delaying study onset. |
|  | “I’ll do it tomorrow” logic | Rationalises postponement with “later/tomorrow” thinking. |
|  | Deadline‑driven bursts | Tends to work in intense bursts close to deadlines rather than steadily. |
|  | Feeling overwhelmed | Experiences the volume, complexity, or density of the course material as overwhelming |
|  |  |  |
|  |  |  |
